# Immune Profile in Patients With COVID-19: Lymphocytes Exhaustion Markers in Relationship to Clinical Outcome

**DOI:** 10.3389/fcimb.2021.646688

**Published:** 2021-04-15

**Authors:** Anna Bobcakova, Jela Petriskova, Robert Vysehradsky, Ivan Kocan, Lenka Kapustova, Martina Barnova, Zuzana Diamant, Milos Jesenak

**Affiliations:** ^1^ Centre for Primary Immunodeficiencies, Clinic of Pneumology and Phthisiology, Jessenius Faculty of Medicine, Comenius University in Bratislava, Martin University Hospital, Martin, Slovakia; ^2^ Department of Clinical Immunology and Allergology, Martin University Hospital, Martin, Slovakia; ^3^ Centre for Primary Immunodeficiencies, Clinic of Pediatrics, Jessenius Faculty of Medicine, Comenius University in Bratislava, Martin University Hospital, Martin, Slovakia; ^4^ Department of Respiratory Medicine and Allergology, Institute for Clinical Science, Skane University Hospital, Lund University, Lund, Sweden; ^5^ Department of Respiratory Medicine, First Faculty of Medicine, Charles University and Thomayer Hospital, Prague, Czechia

**Keywords:** SARS-CoV-2, COVID-19, immune cells exhaustion, clinical outcome, immunologic predictors

## Abstract

The velocity of the COVID-19 pandemic spread and the variable severity of the disease course has forced scientists to search for potential predictors of the disease outcome. We examined various immune parameters including the markers of immune cells exhaustion and activation in 21 patients with COVID-19 disease hospitalised in our hospital during the first wave of the COVID-19 pandemic in Slovakia. The results showed significant progressive lymphopenia and depletion of lymphocyte subsets (CD3^+^, CD4^+^, CD8^+^ and CD19^+^) in correlation to the disease severity. Clinical recovery was associated with significant increase in CD3^+^ and CD3^+^CD4^+^ T-cells. Most of our patients had eosinopenia on admission, although no significant differences were seen among groups with different disease severity. Non-survivors, when compared to survivors, had significantly increased expression of PD-1 on CD4^+^ and CD8^+^ cells, but no significant difference in Tim-3 expression was observed, what suggests possible reversibility of immune paralysis in the most severe group of patients. During recovery, the expression of Tim-3 on both CD3^+^CD4^+^ and CD3^+^CD8^+^ cells significantly decreased. Moreover, patients with fatal outcome had significantly higher proportion of CD38^+^CD8^+^ cells and lower proportion of CD38^+^HLA-DR^+^CD8^+^ cells on admission. Clinical recovery was associated with significant decrease of proportion of CD38^+^CD8^+^ cells. The highest AUC values within univariate and multivariate logistic regression were achieved for expression of CD38 on CD8^+^ cells and expression of PD1 on CD4^+^ cells alone or combined, what suggests, that these parameters could be used as potential biomarkers of poor outcome. The assessment of immune markers could help in predicting outcome and disease severity in COVID-19 patients. Our observations suggest, that apart from the degree of depletion of total lymphocytes and lymphocytes subsets, increased expression of CD38 on CD3^+^CD8^+^ cells alone or combined with increased expression of PD-1 on CD3^+^CD4^+^ cells, should be regarded as a risk factor of an unfavourable outcome in COVID-19 patients. Increased expression of PD-1 in the absence of an increased expression of Tim-3 on CD3^+^CD4^+^ and CD3^+^CD8^+^ cells suggests potential reversibility of ongoing immune paralysis in patients with the most severe course of COVID-19.

## Introduction

In 2020, SARS-CoV-2 virus has spread all around the world, causing COVID-19 disease in over 70 million of people worldwide by December 2020. While in some patients the course of this infection may be mild or even asymptomatic, for many others, especially older patients with chronic diseases, it may be life-threatening or even fatal (Chen N. et al., 2020; Lu et al., 2020; Madjid et al., 2020; Song et al., 2020; Wang D. et al., 2020). As the worsening of the clinical state, associated with the development of change to: hyperinflammatory syndrome (cytokine storm), may be sudden, from the beginning of this pandemic, clinicians and scientists have been trying to identify potential predictors of the severity of the disease and disease outcome in individual patients. This could help us to identify patients requiring special care: i.e., early ICU admission, intense monitoring, more aggressive and once available, more specific/targeted therapy.

Up to now, increased serum inflammatory markers (CRP and procalcitonin), cardiac specific markers (CK-MB, troponin), coagulation (D-Dimer), and some enzymes (ASAT, ALAT, γ-GT, LDH) as well as selected parameters of complete blood count (lymphocytes, thrombocytes, eosinophils) are recognized to be associated with severe COVID-19 disease (Danwang et al., 2020).

More recently, immune profiling in SARS-CoV-2 infected patients has raised the interest in some other potential biomarkers, which could serve as new treatment targets. Proven dysregulations of the immunity could justify immune intervention in patients at high risk of poor outcome (Jesenak et al., 2020).

Specific cellular immunity including both CD4^+^ and CD8^+^ T-cells is crucial for achieving control over viral infections. Although immune system is important for disease elimination, its over-activation may cause significant harm and contribute to disease pathogenesis. Differences in immune profiles can help to better understand the pathogenesis and clinical expression of COVID-19 (Song et al., 2020).

In this observational study, we aimed to analyse the changes in selected immune parameters in relationship to various degrees of COVID-19 severity and clinical outcomes. Specifically, we were interested in markers of immune cells exhaustion and activation.

## Patients and Methods

This was a single-centre, retrospective observational study including hospitalized adult patients with SARS-CoV-2 infection. The study was approved by the local Ethical Committee (Decision No. EK UNM 77/2020).

We retrospectively analysed the immune profile of 21 adult patients with COVID-19 hospitalized in our hospital (Martin University Hospital, Martin city, Slovakia) between April 2020 and August 2020 (**Table 1**). All patients were RT-PCR confirmed positive for SARS-CoV-2 retrieved from nose and throat swabs. Patients were divided into 4 groups according to the severity of their COVID-19 disease: Group A (including asymptomatic patients or patients with mild/moderate symptoms, who were hospitalized for another reason than COVID-19, did not have signs of pneumonia on chest X-ray and did not require supplementation of oxygen); Group B (including patients with severe symptoms, signs of bilateral pneumonia on chest X-ray and/or SpO2 below 93% on room air, thus requiring supplementation of oxygen); Group C (including critically ill patients hospitalized in intensive care unit with need of HFNO, non-invasive ventilation support or mechanical ventilation) and Group D (including patients who died as a result of COVID-19). In the final analysis, we did not include one asymptomatic patient, who received systemic corticosteroids as anti-oedematous therapy for a brain tumour, as this therapy could affect blood cell counts/differentials and possibly also lymphocyte subsets.

**Table 1:** Clinical characteristics of patients included in the study.

	Age range	Patients’ characteristics and comorbidities	Therapy
**GROUP A (n=4; patients with asymptomatic, mild/moderate COVID-19, without signs of pneumonia on chest X-ray, without the need of oxygen supplementation)**			
**1**	50-54	hypertension, hypothyreosis, chronic ischemic heart disease	inosine pranobex, betaglucans
**2**	60-64	chronic ischemic heart disease, schizophrenia, Parkinson’s disease	betaglucans, antibiotics
**3**	90-94	hypertension, hypothyreosis, chronic ischemic heart disease, acute cholangitis	antibiotics
4*	35-39	brain tumour, seizure	inosine pranobex, zinc
**GROUP B (n=10; severe symptoms, bilateral pneumonia on chest X-ray, SpO2 below 93% on room air, thus requiring oxygen supplementation)**			
**1**	50-54	hypertension	inosine pranobex, antibiotics, zinc
**2**	45-49	supraventricular tachycardia	inosine pranobex, antibiotics, zinc
**3**	85-89	hypertension, chronic kidney disease, B-NHL, history of breast cancer and basocellular carcinoma (in remission)	betaglucans, inosine pranobex, antibiotics
**4**	45-49	persistent atrial fibrillation	inosine pranobex, antibiotics
**5**	65-69	hypertension	antibiotics
**6**	65-69	hypertension, chronic ischemic heart disease, diabetes type 2, COPD	antibiotics, betaglucans, inosine pranobex,
**7**	65-69	hypertension, chronic ischemic heart disease, diabetes type 2, atrial fibrillation	zinc, inosine pranobex, vitamin D, betaglucans, antibiotics
**8**	50-54	no chronic comorbidities	betaglucans, inosine pranobex
**9**	45-49	hypothyreosis	antibiotics
**10**	50-54	hypertension, hepatopathy, sticky platelet syndrome	antibiotics
**GROUP C (n=3; critically ill patients requiring intensive care unit stay and either invasive ventilation or non-invasive ventilation support or HFNO)**			
**1**	75-79	COPD, hypertension	antibiotics, azithromycin + hydroxychloroquine, lopinavir/ritonavir + ribavirine, IVIG, betaglucans, transfer factor, azoximer bromide
**2**	75-79	hypertension, COPD, seizures, Parkinson’s disease, history of stroke, urosepsis	betaglucans, azoximer bromide, inosine pranobex, antibiotics, IVIG
**3**	85-89	chronic ischemic heart disease, hypertension, rheumatoid arthritis (untreated)	antibiotics
**GROUP D (n=4; deceased patients)**			
**1**	85-89	mixed dementia	azithromycin + hydroxychloroquine, antibiotics, azoximer bromide
**2**	90-94	hypertension, chronic ischemic heart disease, myasthenia gravis, chronic kidney disease, history of stroke	azithromycin + hydroxychloroquine, inosine pranobex, azoximer bromide, antibiotics
**3**	70-74	hypertension, diabetes type 2, history of colorectal adenocarcinoma (in remission)	antibiotics, azithromycin + hydroxychloroquine, azoximer bromide
**4**	80-84	hypertension, chronic ischemic heart disease, permanent atrial fibrillation, history of stroke	inosine pranobex, betaglucans, azithromycin + hydroxychloroquine, antibiotics

*Patient No. 4 in group A was not included in the final analysis (treated with systemic corticoids as antiedematous therapy, that could potentially affect results).

HFNO, high flow nasal oxygenotherapy; IVIG, intravenous immunoglobulins; COPD, chronic obstructive pulmonary disease; B-NHL, B-cell non-Hodgkin’s lymphoma.

**Figure 1:**
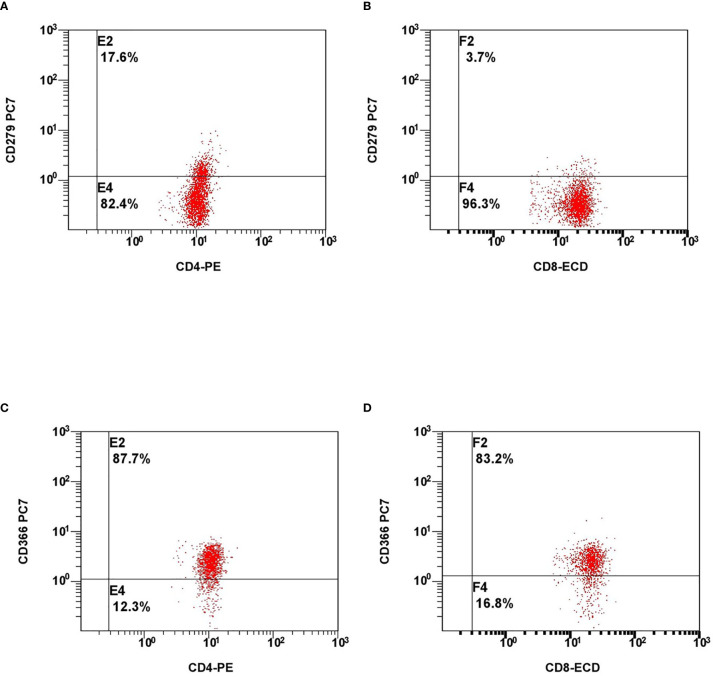
Representative flow cytometry plots of expression of lymphocyte exhaustion markers. Expression of PD-1 on CD4+ cells **(A)**, CD8+ cells **(B)** and expression of Tim-3 on CD4+ cells **(C)**, CD8+ cells **(D)**.

The analysed immune profile on hospital admission consisted of differential blood cell count, immunoglobulins (IgG, IgA, IgM, IgE), complement components (C3 and C4), and subtyping of lymphocytes by flow cytometry (on admission and on weekly basis). Subtyping of lymphocytes included the basic panel (CD3^+^, CD19^+^, CD4^+^, CD8^+^, CD16^+^56^+^), phenotyping of B-cell subsets, markers of cells exhaustion (PD-1 and Tim-3 on CD4^+^ and CD8^+^ cells (**Figures 1A–D**), NKG2A (CD159) on CD8^+^ and CD16^+^56^+^ cells) and markers of cell activation (HLA-DR on CD3^+^ cells, CD38 and CD38 and HLA-DR co-expression on CD8^+^ cells).

Statistical analysis was performed using MedCalc statistical software (MedCalc^®^ Statistical Software version 19.5.3 (MedCalc Software Ltd, Ostend, Belgium; https://www.medcalc.org; 2020). As groups A, C and D included only 3 to 4 patients, we could not effectively evaluate the normality of distribution in each of groups. For this reason, we have chosen non-parametric versions of statistical tests to analyse our results (Kruskal-Wallis test with post-hoc analysis Conover test, Mann-Whitney test for independent samples, Wilcoxon test for paired samples). To test, weather examined markers of lymphocyte activation and exhaustion could be considered as independent risk factors for poor outcome in COVID-19 patients, univariate and multivariate logistic regression with calculation of AUC were used.

## Results

Altogether, 20 patients were selected for the final analysis. They were divided into 4 groups: Group A – 3 patients with asymptomatic, mild to moderate COVID-19, without signs of pneumonia on chest X-ray, without need of oxygen supplementation; Group B – 10 patients with severe symptoms, bilateral pneumonia on chest X-ray, SpO2 below 93% on room air, requiring oxygen supplementation; Group C – 3 critically ill patients requiring intensive care unit stay and either invasive ventilation of non-invasive ventilation support or HFNO; Group D – 4 deceased patients.

**Figure 2:**
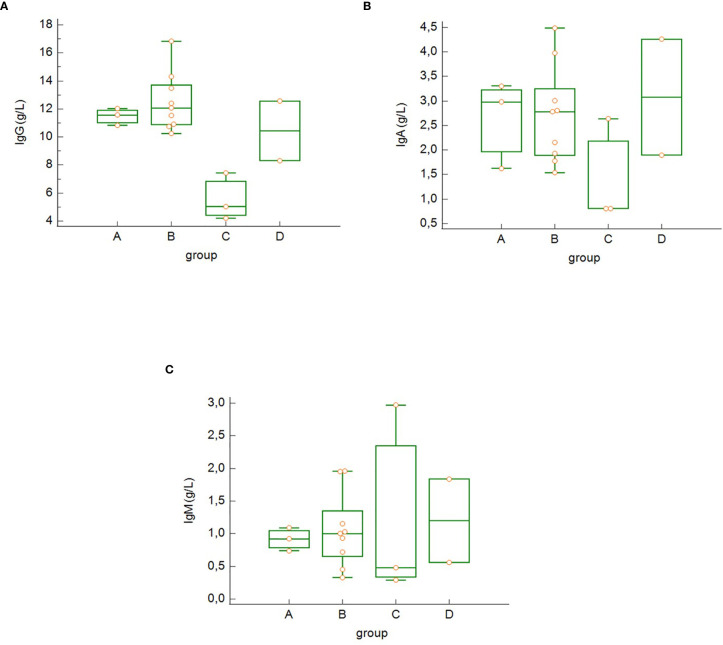
Differences in the serum concentration of IgG **(A)**, IgA **(B)** and IgM **(C)** among the groups of COVID-19 patients (differences not significant – Kruskal-Wallis test). Group A: n = 3 patients, Group B: n = 10 patients, Group C: n = 3 patients, Group D: n = 4 patients.

As anticipated, the different clinical subsets according to COVID-19 severity (groups A–D) were characterized by different changes in immune biomarkers and lymphocytes subsets. We found decreased concentrations of serum IgG (**Figure 2A**), IgA (**Figure 2B**), but not IgM (**Figure 2C**) in the group of critically ill patients, who survived COVID-19 (group C) compared to the other groups; however, these differences were not statistically significant. There were no significant differences in serum concentrations of C3 and C4 among the four severity groups in our study.

**Figure 3:**
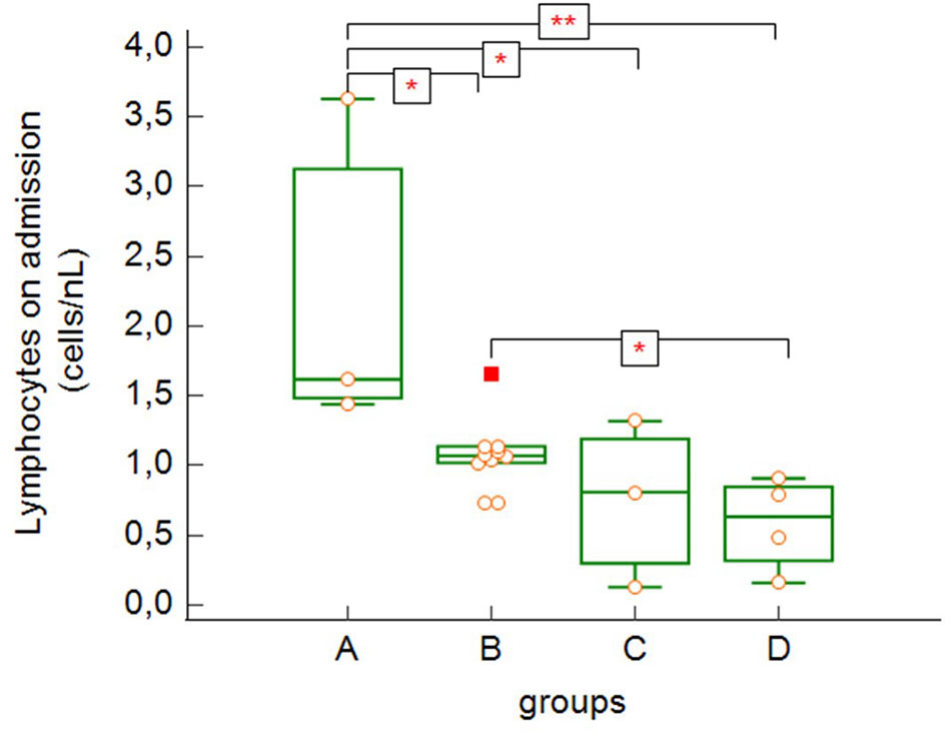
Absolute lymphocyte counts on admission to the hospital (group A versus group B: p = 0.043, group A versus group C: p = 0.038, group A versus group D: p = 0.007, group B versus group D: p = 0.043 – Kruskal-Wallis test, post-hoc analysis Conover test). Group A: n = 3 patients, Group B: n = 10 patients, Group C: n = 3 patients, Group D: n = 4 patients. *p < 0.05, **p < 0.01.

The total lymphocyte count on admission (**Figure 3**) was significantly higher in asymptomatic patients/patients with mild to moderate symptoms (group A) compared to the more severe groups of patients (B, C and D) (group A versus group B: p = 0.043, group A versus group C: p = 0.038, group A versus group C: p = 0.007) as well as in the group of severe patients (B) compared to the deceased patients (group D) (group B versus group D: p = 0.043).

**Figure 4:**
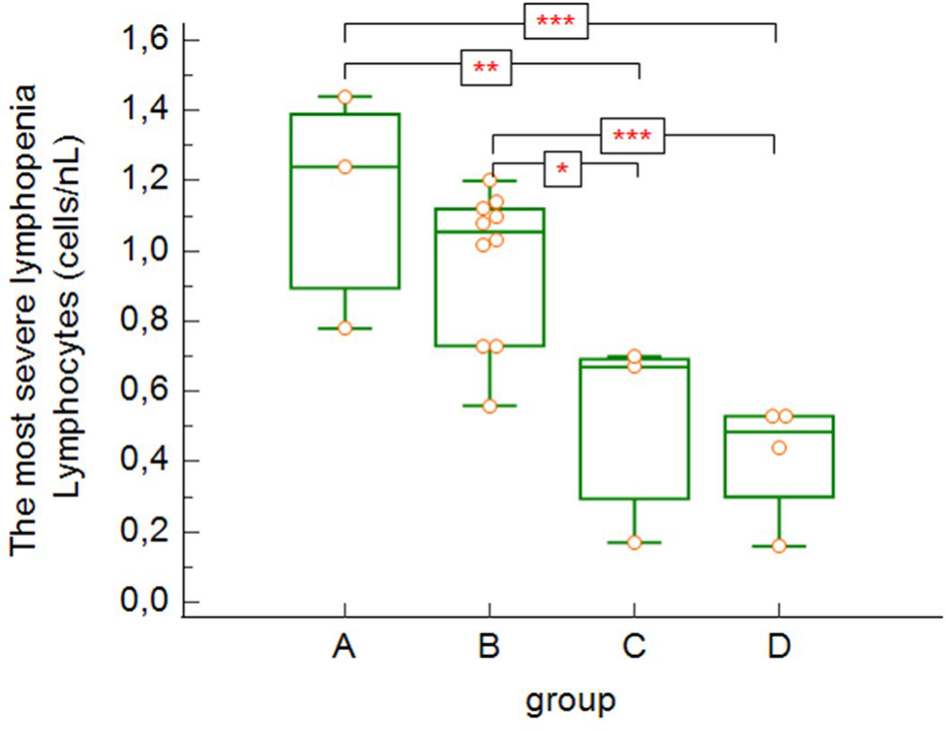
The lowest absolute lymphocytes counts found during hospitalization (group A versus group C: p = 0.004, group A versus group D: p = 0.0009, group B versus group C: p = 0.0103, group B versus group D: p = 0.0009 – Kruskal-Wallis test, post-hoc analysis Conover test). Group A: n = 3 patients, Group B: n = 10 patients, Group C: n = 3 patients, Group D: n = 4 patients. *p < 0.05, **p < 0.01, ***p < 0.001.

The most severe lymphopenia during hospitalization (**Figure 4**) was observed in the most severe groups of patients – critically ill and deceased patients (groups C and D), with significant differences between groups A, B and C, D (group A versus group C: p = 0.004, group A versus group D: p = 0.0009, group B versus group C: p = 0.010, group B versus group D: p = 0.0009).

**Figure 5:**
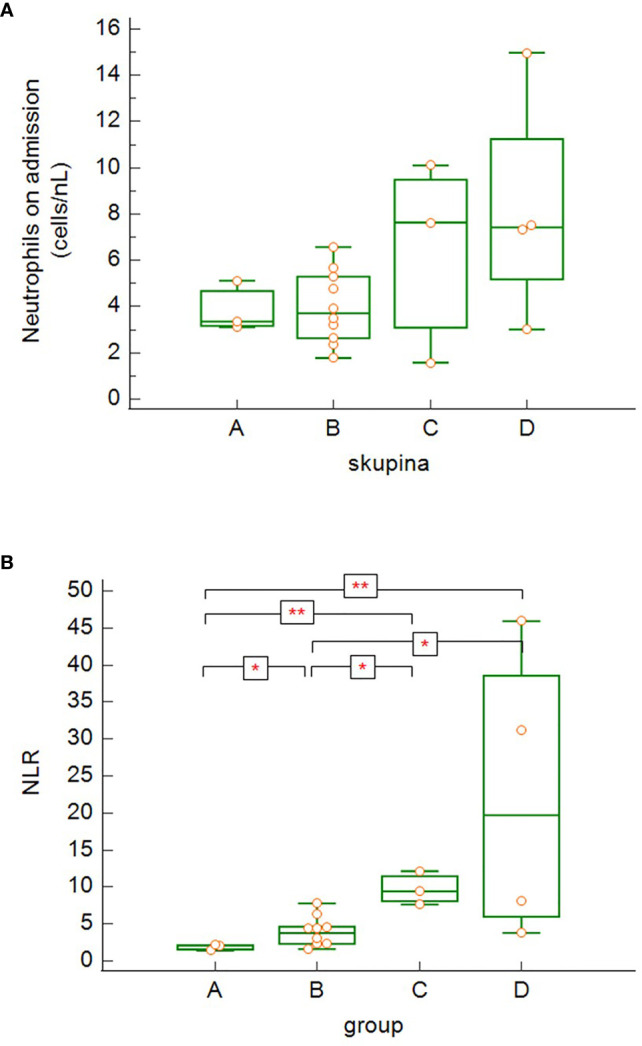
The differences in absolute neutrophil counts **(A)** and neutrophil-to-lymphocyte ratio (NLR) **(B)** among the patients’ groups on admission (4A – differences not significant − Kruskal-Wallis test; 4B – group A versus group B: p = 0.029, group A versus group C: p = 0.0014, group A versus group D: p = 0.0014; group B versus group C: p = 0.014, group B versus group D: p = 0.012 − Kruskal-Wallis test, post-hoc analysis Conover test). Group A: n = 3 patients, Group B: n = 10 patients, Group C: n = 3 patients, Group D: n = 4 patients. *p < 0.05, **p < 0.01.

Although there was a slightly increasing tendency in the neutrophil count on admission correlating with the severity of COVID-19 disease (**Figure 5A**), it was not statistically significant. On the other hand, we noticed significant differences in neutrophil-to-lymphocyte ratio (NLR) on admission in relation with disease severity (**Figure 5B**). NRL was significantly lower in asymptomatic/mild to moderate patients (group A) compared to other groups with more severe COVID-19 disease (groups B, C, D) (group A versus group B: p = 0.029, group A versus group C: p = 0.001, group A versus group D: p = 0.001) and significantly lower in the group of patients with severe disease (B) compared to critically ill or deceased patients (group B versus group C: p = 0.014, group B versus group D: p = 0.012).

There were no significant differences in total eosinophil count on admission among our groups, however, eosinopenia was common and the majority of patients (70%) had an absolute level of eosinophils below normal (< 0.03/nL) on admission.

**Figure 6:**
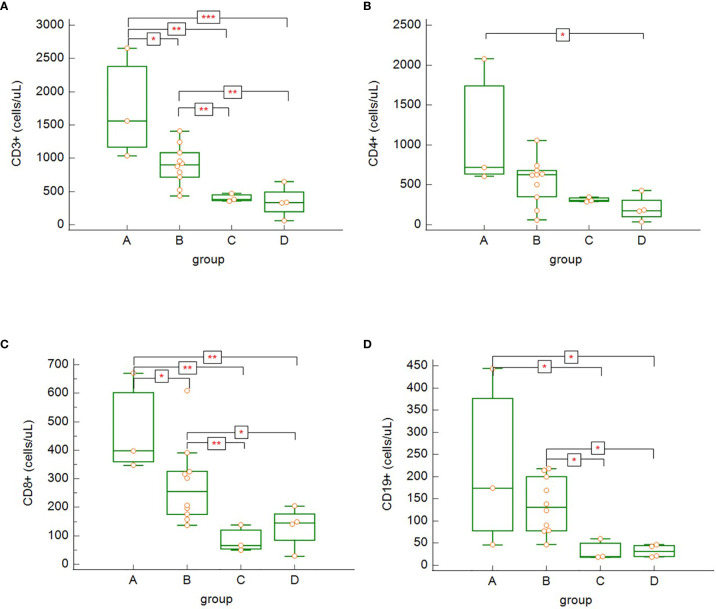
The differences in absolute counts of CD3^+^ **(A)** – group A versus group B: p = 0.035, group A versus group C: p = 0.0012, group A versus group D: p = 0.0004, group B versus group C: p = 0.009, group B versus group D: p = 0.0012; CD3^+^CD4^+^ **(B)** – group A versus group D: p = 0.036; CD3^+^CD8^+^ **(C)** – group A versus group B: p = 0.043, group A versus group C: p = 0.002, group A versus group D: p = 0.004, group B versus group C: p = 0.007, group B versus group D: p = 0.033; and CD19^+^ **(D)** – group A versus group C: p = 0.017, group A versus group D: p = 0.017, group B versus group C: p = 0.011, group B versus group D: p = 0.011, among the patients’ groups on admission. Kruskal – Wallis test, post-hoc analysis Conover test). Group A: n = 3 patients, Group B: n = 10 patients, Group C: n = 3 patients, Group D: n = 4 patients. *p < 0.05, **p < 0.01, ***p < 0.001.

Our data show significant differences in lymphocytes subsets across the different severity groups (A-D). In general, we observed decreasing tendency of all lymphocytes subsets in relation with increasing disease severity. CD3^+^ (**Figure 6A**) and CD3^+^CD8^+^ (**Figure 6C**) cells were significantly higher in asymptomatic patients/patients with mild to moderate symptoms (group A) compared to other groups (B, C and D) (count of CD3^+^: group A versus group B: p = 0.035, group A versus group C: p = 0.001, group A versus group D: p = 0.0004; count of CD8^+^: group A versus group B: p = 0.043, group A versus group C: p = 0.002, group A versus group D: p = 0.004) and significantly higher in patients with severe COVID-19 disease (B) compared to critically ill patients (group C) or patients, who died (group D) (count of CD3^+^: group B versus group C: p = 0.0097, group B versus Group D: p = 0.001; count of CD8^+^: group B versus group C: p = 0.007, group B versus group D: p = 0.033). CD3^+^CD4^+^ cells (**Figure 6B**) were significantly higher in low symptoms group (group A) compared to perished patients (group D) (group A versus group D: p = 0.036). Although there was clearly decreasing trend of the total count of CD3^+^CD4^+^ from group A towards group D, statistical significance of the differences between low symptom group (A) versus ICU group (Group C; p = 0.06) and patients with severe COVID-19 (Group B) compared to deceased patients (Group D; p = 0.059) was only borderline. The lack of CD19^+^ (**Figure 6D**) cells was significantly higher in two most severe groups of patients (groups C and D) compared to less severe groups (A and B) (group A versus group C: p = 0.017, group A versus group D: p = 0.017, group B versus group C: p = 0.011, group B versus group D: p = 0.011). Although patients with asymptomatic/oligosymptomatic COVID-19 (group A) had higher NK cell count compared to other groups, the difference was not statistically significant.

**Figure 7:**
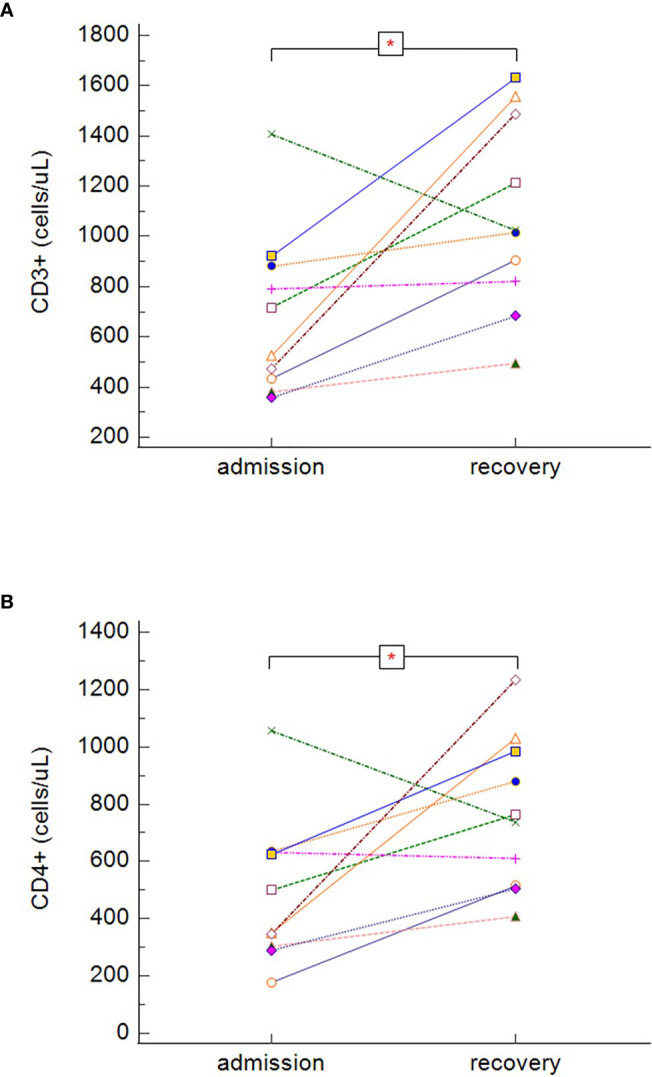
The dynamics of absolute counts of CD3^+^ **(A)** and CD3^+^CD4^+^ **(B)**, in symptomatic surviving patients during the hospitalization (dynamics of CD3^+^: p = 0.022, dynamics of CD3^+^CD4^+^: p = 0.037 − Wilcoxon test for paired samples). Group B + C: n = 13 patients. *p < 0.05.

During recovery, survivors with severe or critical course of COVID-19 (groups B and C) had significantly increasing total number of CD3^+^ cells (**Figure 7A**) (p = 0.022) and CD3^+^CD4^+^ cells (**Figure 7B**) (p = 0.037), however, no statistically significant differences were found in the dynamics among other lymphocytes subsets. As we obtained only one paired sample in the group of non-survivors (patients died before re-tests), it was not possible to effectively estimate the dynamics of the lymphocyte subsets in this group (group D).

**Figure 8:**
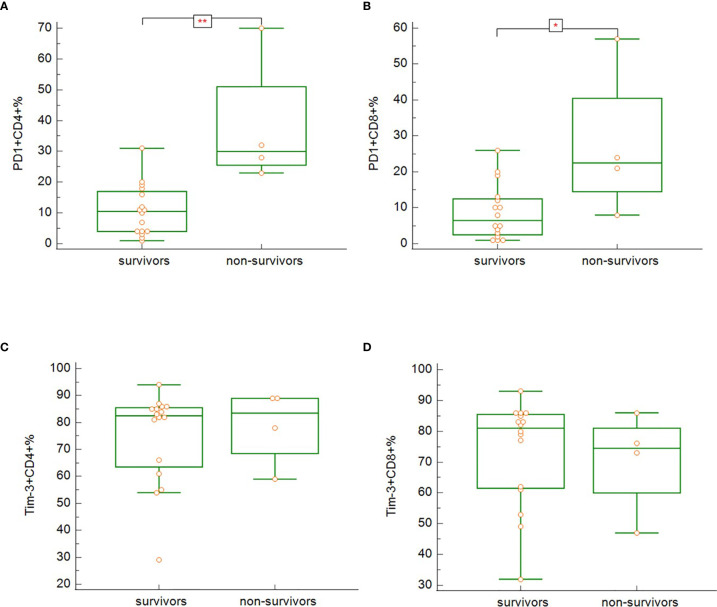
The differences in expression of PD-1 on CD3^+^CD4^+^ (**A**; p = 0.005) and CD3^+^CD8^+^ (**B**; p = 0.033) and Tim-3 on CD3^+^CD4^+^ (**C**; p = n.s.) and CD3^+^CD8^+^ (**D**; p = n.s.) between the survivors and non-survivors at admission. Mann-Whitney test for independent samples. Group A + B + C (i.e. survivors): n = 16 patients, Group D (i.e. non-survivors): n = 4 patients. *p < 0.05, **p < 0.01.

**Figure 9:**
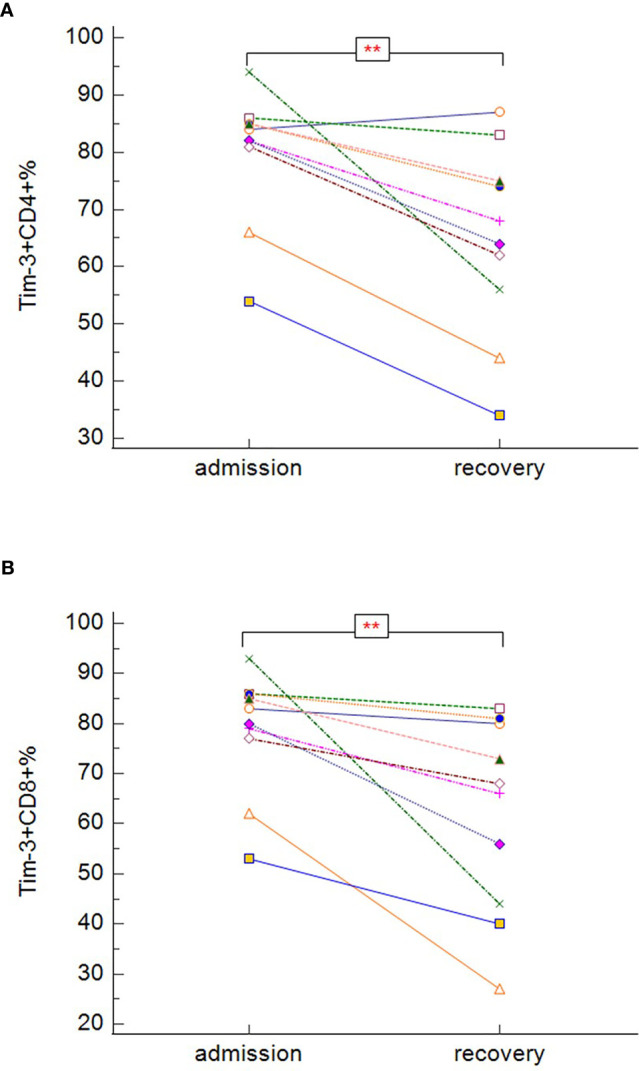
The changes in expression of Tim-3 on CD3^+^CD4^+^ **(A)** and CD3^+^CD8^+^ **(B)** during the hospitalization in symptomatic recovered patients (Tim-3 on CD3^+^CD4^+^: p = 0.008; Tim-3 on CD3^+^CD8^+^: p = 0.005 − Wilcoxon test for paired samples). Group B + C: n = 13 patients. **p < 0.01.

In our study, survivors (groups A, B, C), regardless of the severity of the disease, had significantly lower expression of PD-1 on both CD3^+^CD4^+^ (**Figure 8A**) (p = 0.005), and CD3^+^CD8^+^ (**Figure 8B**) (p = 0.033) cells. No differences between survivors and non-survivors were found in Tim-3 expression on CD3^+^CD4^+^ (**Figure 8C**) nor CD3^+^CD8^+^ (**Figure 8D**) cells. During recovery in severe and critically ill patients (groups B and C), Tim-3 expression on both CD3^+^CD4^+^ (**Figure 9A**) (p = 0.008) and CD3^+^CD8^+^ (**Figure 9B**) (p = 0.005) cells was significantly decreasing.

**Figure 10:**
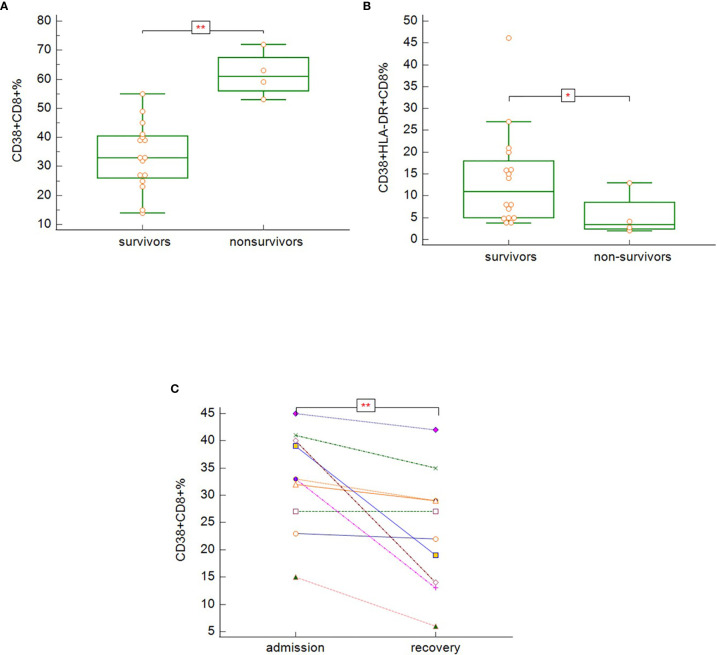
The differences in expression of CD38 on CD3^+^CD8^+^ (**A**; p = 0.003), co-expression of CD38 and HLA-DR on CD3^+^CD8^+^ (**B**; p = 0.037) − Mann-Whitney test for independent samples, Group A + B + C (i.e. survivors): n = 16 patients, Group D (i.e. non-survivors): n = 4 patients; and dynamics of CD38 expression on CD3^+^CD8^+^ during hospitalization among the symptomatic survivors (**C**; p = 0.008) − Wilcoxon test for paired samples, Group B + C: n = 13 patients.

Non-survivors (group D) had significantly higher proportion of CD3^+^CD8^+^ cells expressing CD38 activation marker (**Figure 10A**) (p = 0.003) and significantly lower proportion of CD3^+^CD8^+^ cells co-expressing both CD38 and HLA-DR (**Figure 10B**) (p = 0.037). There was no significant difference in the expression of HLA-DR on total T-cells. In severe and critically ill patients, who survived (groups B and C), there was decreasing tendency of proportion of CD38^+^CD8^+^ cells during recovery (**Figure 10C**) (p = 0.008). It was not possible to assess the dynamics of T-cells expressing activation markers CD38 and HLA-DR in the non-survivors due to the reason described in the previous section.

**Figure 11:**
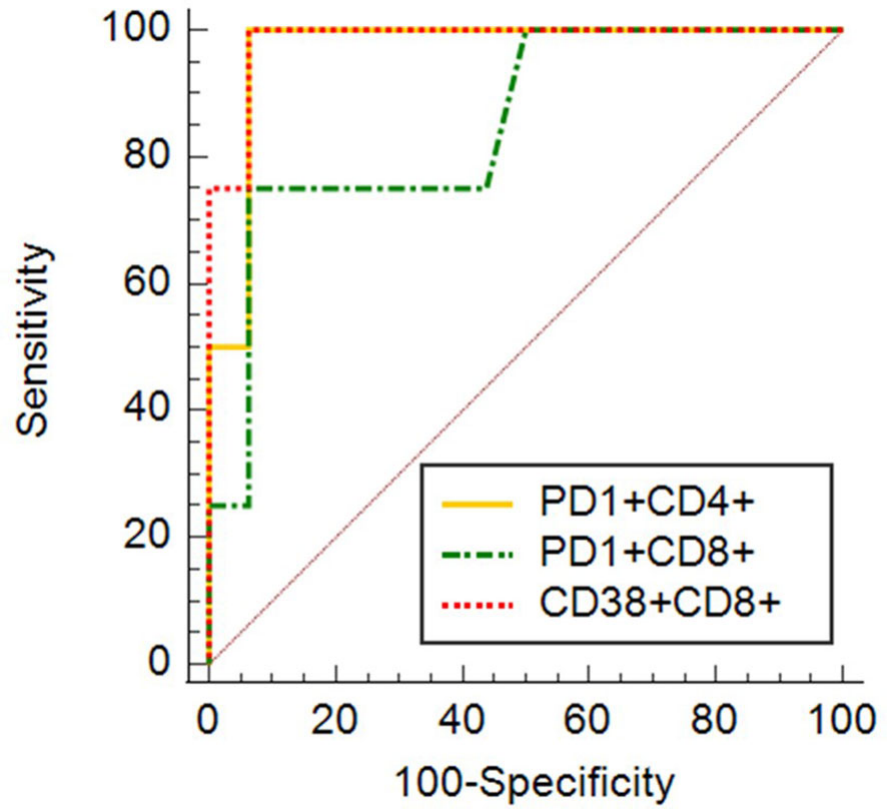
ROC curves for expression of CD38 on CD8^+^ (AUC 0.984) cells, expression of PD1 on CD4^+^ cells (AUC 0.969) and expression of PD1 on CD8^+^ cells (AUC 0.852).

To confirm, weather mentioned markers of immune cell activation and exhaustion could potentially be used as markers of COVID-19 outcome in relation to survival, additionally to standard testing, we proceeded with univariate (for one variable) and multivariate (for several variables) logistic regression. AUC, derived from ROC curve, is the measure of the ability of a classifier to distinguish between classes. The maximum of AUC value is 1, when all samples are truly assigned into the groups. An AUC of 0.5 is equivalent to randomly classifying subjects as either positive or negative (the classifier is of no practical utility). The highest AUC values were obtained for expression of CD38 on CD8^+^ (AUC 0.984) cells, expression of PD1 on CD4^+^ cells (AUC 0.969) and expression of PD1 on CD8^+^ cells (AUC 0.852) (**Figure 11**). AUC values for other examined markers were too low (close to 0.5). Combination of expression of CD38 on CD8^+^ with expression of PD1 on CD4^+^ cells has shown promising results as an ideal biomarker in our patients’ population (AUC 1.0).

Except significantly higher proportion of switched memory cells in A and D groups (p = 0.039), no significant differences between different severity groups were found in other examined parameters (subtypes of B-cells, CD4^+^CD45^+^RO^+^ T-cells, expression of CD159 (NKG2A) on CD3^+^CD8^+^ and NK cells).

## Discussion

Lymphopenia is one of major laboratory findings in patients with COVID-19, with both diagnostic and prognostic potential (Terpos et al., 2020). Dysregulated immune responses participate in COVID-19 pathogenesis (Quin et al., 2020). Our results demonstrate significant progressive lymphopenia and increasing NLR (on admission) with increasing disease severity, which is in accordance with already published data (Danwang et al., 2020; Huang et al., 2020; Liu Y. et al., 2020; Ma et al., 2020). Although the majority of our patients had eosinopenia on admission, there was no significant difference across the severity groups. A meta-analysis of 1289 COVID-19 cases found a significant correlation between elevated leucocytes and decreased lymphocytes counts in relation to the disease severity. In published studies, the majority of severe cases presented on admission with a total lymphocyte count <1 **×** 10^9^/L, while non-severe cases tended to have lymphocytes above this level (Huang et al., 2020). Similar findings, regarding haematological parameters, were reported in a meta-analysis by Danwang et al.: severe COVID-19 cases had a significantly lower lymphocyte count, proportion of lymphocytes, CD3^+^ cells, eosinophils and a significantly higher neutrophil count. Non-survivors had significantly lower level of lymphocytes and higher level of white blood cells (Danwang et al., 2020). Higher neutrophil-to-lymphocyte ratio was shown to be associated with deterioration and mortality in COVID-19 patients (Liu Y. et al., 2020; Ma et al., 2020). Comparing severe and non-severe cases, Chen R. et al. (2020) showed significantly higher leucocyte and neutrophil count, significantly higher neutrophil-to-lymphocyte ratio, lower lymphocyte count, lower percentages of monocytes, eosinophils and basophils in severe cases (Chen R. et al., 2020).

Although it remains to be clarified what underlying mechanism is responsible for lymphopenia in COVID-19 patients, several authors speculate about increased apoptosis, inhibition or down-regulation of lymphocytes induced by cytokines, metabolic disorders (e.g., lactate acidosis) and increased glucocorticoid levels. Even direct infection and destruction of lymphocytes and lymphatic tissue by virus has been suggested (Danwang et al., 2020; Huang et al., 2020; Tan et al., 2020; Terpos et al., 2020; Xu et al., 2020). Massive migration of lymphocytes to the lungs may be partially accountable for this observation (Song et al., 2020).

Although lymphopenia is an important laboratory marker of SARS-CoV-2 infection, however, especially during the influenza season, clinicians should be aware, that lymphopenia is not specific for COVID-19 and can be seen in other viral pneumonias as well (Leroy et al., 2001; Bellelli et al., 2019; Danwang et al., 2020). Eosinopenia, although associated with COVID-19 disease, is neither specific for this disease, as it was also observed in other viral diseases, e.g. severe RSV infection (Pinto et al., 2006).

Several studies reported on changes in lymphocytes subsets in COVID-19. While CD3^+^CD8^+^ cells are vital for the elimination of virus infected cells by secretion of perforins, granzymes and interferons (Mescher et al., 2006), CD3^+^CD4^+^ cells participate by co-stimulating effect on CD3^+^CD8^+^ and CD19^+^ B-cells (Zhu et al., 2009). We found a significant decline in CD3^+^, CD19^+^, CD3^+^CD4^+^ and CD3^+^CD8^+^ cells correlating with increasing disease severity, while on the other hand, there was only a non-significant, decreasing trend of NK cells in relation to the disease severity. Published data of several authors agree with the finding of increasing decline in CD3^+^, CD3^+^CD4^+^ and CD3^+^CD8^+^ cells in relation to the disease severity (Chen R. et al., 2020; Diao et al., 2020; Song et al., 2020; Wang F. et al., 2020; Jiang M. et al., 2020; Liu R. et al., 2020). In contrast, results concerning CD19^+^ cells and NK cells are not consistent. Urra et al. (2020) compared T-lymphocytes subsets in ICU and non-ICU patients and noticed only significant difference in the number of CD3^+^CD8^+^ cells, which was lower in the ICU group and correlated with increased concentrations of inflammatory mediators (Urra et al., 2020). In addition to these findings, Diao et al. (2020) observed an age-dependent decline in T-cells (the most severe reduction in patients over 60 years), providing a possible explanation for the increased susceptibility for COVID-19 and its severe course in senior individuals (Diao et al., 2020). Immune depression related to pre-existing chronic disease could be considered as a risk factor for SARS-CoV-2 infection and its severity as well (Applegate and Ouslander, 2020). Enhancing T-cell immunity might prevent progression of severe patients to critical illness (Liu R. et al., 2020).

Clinical recovery in our study was associated with significant increase in CD3^+^ and CD3^+^CD4^+^ cells. Jiang Y. et al. (2020) also described significant increase in total lymphocytes, CD3^+^ and CD3^+^CD8^+^ cells and increasing tendency, hence non-significant, in NK cells (Jiang Y. et al., 2020). Similar to our results, Diao et al. (2020) reported increasing levels of not only CD3^+^, CD3^+^CD4^+^ cells, but CD3^+^CD8^+^ as well and have shown, that these changes are associated with decreasing concentrations of several cytokines (TNF-α, IL-6 and IL-10) – suggesting possible effect of these cytokines on T-cell survival and proliferation in COVID-19 patients (Diao et al., 2020). Jiang M. et al. (2020) pointed to the recovery of CD3^+^, CD3^+^CD4^+^ and CD3^+^CD8^+^ cell counts in patients with conversion to negative virus nucleic acid test, while there was no significant difference in the dynamics of these cells count in patients with persistent nucleic acid test positivity (Jiang M. et al., 2020).

While we found a significantly higher proportion of CD38^+^CD8^+^ and significantly lower proportion of CD38^+^HLA-DR^+^CD8^+^ cells in non-survivors, Song et al. (2020), in contradiction to our findings, described significantly higher proportion of CD38^+^HLA-DR^+^CD8^+^ in severe compared to mild cases, there was no significant difference in the proportion of CD38^+^CD8^+^ cells between mild and severe cases in Song’s study (Song et al., 2020). Jiang Y. et al. (2020) described increased proportion of CD38^+^CD8^+^ cells and HLA-DR^+^CD8^+^ cells in COVID-19 patients compared to healthy controls, although no significant differences were found among different disease severity groups (Jiang Y. et al., 2020). Our results suggest decreasing expression of only CD38 on CD3^+^CD8^+^ cells during recovery. In Jiang’s study (Jiang Y. et al., 2020), there was non-significantly decreasing trend of both CD38 and HLA-DR expression on CD3^+^CD8^+^ cells (Jiang Y. et al., 2020). In a case report, Thevarajan et al. (2020) described a rapid increase in the co-expression of CD38 and HLA-DR on CD3^+^CD8^+^ cells before clinical improvement (Thevarajan et al., 2020). Quin et al. (2020) compared proportion of HLA-DR^+^CD8^+^ in severe and non-severe patients, but no significant difference was found (Quin et al., 2020). Regarding expression of activation markers on lymphocytes subsets, Wang F. et al. (2020) pointed to significantly increased expression of HLA-DR on both CD3^+^CD4^+^ and CD3^+^CD8^+^ cells in severe and extremely severe patients compared to mild cases. Hyperactivation of CD3^+^CD4^+^ and CD3^+^CD8^+^ cells due to persistent and excessive inflammatory responses may lead to the development of more severe disease in SARS-CoV-2 infected patients and may finally lead to cell exhaustion, anergy and apoptosis in later stages of the disease (Wang F. et al., 2020).

Not only a reduction in absolute numbers of lymphocyte subsets, but also increased expression of the markers of lymphocyte exhaustion, could potentially be responsible for disease progression and unfavourable outcome (Chen R. et al., 2020). In our study, in-hospital death was associated with significantly higher proportion of both PD-1^+^CD3^+^CD4^+^ and PD-1^+^CD3^+^CD8^+^ cells, but there was no significant difference among different severity groups of survivors. In addition, we did not find any significant difference in Tim-3 expression on CD3^+^CD4^+^ nor CD3^+^CD8^+^ cells between survivors and non-survivors. This let us speculate about possibly reversible immune paralysis, which could be potentially averted with early immune modulation intervention. Results of univariate and multivariate logistic regression in our study indicate, that expression of CD38 on CD8^+^ cells alone or together with expression of PD-1 on CD4^+^ cells could be used as a biomarker of survival in hospitalized patients with COVID-19. Although we are aware, that sample size of our groups was too small to make definitive conclusions, these results are encouraging for another bigger studies.

Similarly to our results, Wang F. et al. (2020) also described significant increase in PD-1 expression on CD3^+^CD4^+^ and CD3^+^CD8^+^ cells in extremely severe cases, while only non-significant increasing trend correlating with disease severity was observed in Tim-3 expression (Wang F. et al., 2020). In contrast to our results, Song et al. reported significantly higher expression of both PD1 and Tim-3 on CD3^+^CD8^+^ cells in severe patients compared to those with mild disease, but no significant difference was observed in PD-1 nor Tim-3 expression on CD3^+^CD4^+^ cells (Song et al., 2020). When compared to healthy controls, Diao et al. (2020) noticed increased PD-1 expression on both CD3^+^CD4^+^ and CD3^+^CD8^+^ cells and increased Tim-3 expression on CD3^+^CD4^+^ cells in ICU patients (Diao et al., 2020).

Although no significant difference in NKG2A expression on neither NK nor CD3^+^CD8^+^ cells among different severity groups of COVID-19 patients was found in our study, Zheng M. et al. (2020) published significant increase in NKG2A expression on CD3^+^CD8^+^ and NK cells in COVID-19 patients compared to healthy controls and significant decline in its expression during recovery, what suggests that functional exhaustion of cytotoxic cells might attenuate the effectiveness of antiviral immunity and participate in disease pathogenesis (Zheng M. et al., 2020).

Discrepancies in the above mentioned results concerning immune cell activation and exhaustion may be due to different stages of the infection captured by various authors. It has to be noticed, that mentioned studies focused on comparison of different groups of patients (severe vs. non-severe, survivors vs. non-survivors, COVID-19 patients vs. healthy controls). Moreover, definition of severity varies between different authors. Another factor, that has to be considered, while interpreting results, is small sample size per category in our study, which might be also responsible for bias and reported differences.

Apart from above mentioned markers of immune paralysis, differences in other lymphocytes exhaustion markers were described by several authors as well. Zheng H. Y. et al. (2020) have shown significantly higher expression of TIGIT on CD4^+^ in both mild and severe cases compared to healthy controls and on CD8^+^ cells in patients with severe COVID-19 compared to mild cases and healthy controls, however no significant differences were documented in expression of CTLA-4, PD-1 nor Tim-3 (Zheng H. Y. et al., 2020). In contrary, in a study performed by Herrmann et al. (2020), TIGIT expression on T-cells did not differ significantly in COVID-19 patients compared to healthy controls, however, increased expression of co-inhibitory receptors Tim-3 and LAG-3 was observed on T-cells in both COVID-19 patients versus healthy controls and severe versus mild COVID-19 cases. The same authors observed significantly higher expression of BTLA on transient and effector memory CD8^+^ T-cells in COVID-19 patients versus healthy controls. There were no significant differences in expression of PD-1, nor BTLA between mild and severe cases in this study (Herrmann et al., 2020).

Although there were no significant differences in Tim-3 expression between survivors and non-survivors, recovery of severe and critically ill patients in our study was associated with significant decrease of Tim-3 expression on both CD4^+^ and CD8^+^ cells and, as above mentioned, decrease of CD38 expression on CD8^+^ cells. We did not find significant changes in PD-1 expression dynamics during recovery in these groups. Herrmann et al. (2020) have stated, that co-expression of markers of cell exhaustion depended on the degree of cell activation (HLA-DR, CD38). Recovery of patients with severe COVID-19 in their study was associated with significant decrease of lymphocyte exhaustion markers (Tim-3, PD-1, LAG-3) (Herrmann et al., 2020). The differences in PD-1 expression between our and Herrmann’s study might be related to the small sample sizes in both studies and different definition of severity. Due to lack of pair samples in patients with fatal outcome (death occurred before sample collection), we could not analyze longitudinal development of expression of markers of cell activation nor markers of lymphocyte exhaustion in this group and we can only speculate about possible dynamics of these parameters during disease progression and their differences when compared to recovery. It was presumed, that upregulation of inhibitory molecules during acute infections might represent rather response to excessive immune activation than terminal exhaustion (Legat et al., 2013). We could hypothesize, that improvement in overall clinical condition during recovery will be associated with normalization of lymphocyte hyperactivation and subsequent decrease in inhibitory molecules expression. We think, that decrease of Tim-3 in our survivors with severe and critical disease could be regarded as a marker of recovery, however further studies with bigger sample sizes, containing pair samples from deceased patients are needed to support this hypothesis and to confirm, weather only Tim-3 or other known markers of lymphocyte exhaustion as well, could serve as a predictor of recovery.

Besides oxygen therapy, invasive or non-invasive ventilation support, antipyretics, antibiotics (in patients with suspected or confirmed bacterial superinfection), LMWH, one of our patients received antivirals and 5 patients received combination of hydroxychloroquine with azithromycin (according to local guidelines based on current knowledge to date of sample collection). Moreover, different immune modulators (zinc, vitamin D, betaglucans, inosine pranobex, transfer factor, azoximer bromide, IVIG) were used as supportive treatment. It was suggested that various nutritional supplements (Ferreira et al., 2020; Rao et al., 2020), betaglucans (Ferreira et al., 2020; Murphy et al., 2020; Rao et al., 2020) and transfer factor (Ferreira et al., 2020; Viza et al., 2020) could be used to prevent and treat immune dysregulation induced by SARS-CoV-2. In a study from Czech Republic, the use of inosine pranobex in nursing home residents tested positive for SARS-CoV-2 was associated with significant decrease in case-fatality rate compared to residents who did not receive inosine pranobex (Beran et al., 2020). An observational open label study demonstrated safety of azoximer bromide use in hospitalized patients with COVID-19 (NCT04542226), the results of a multicentre prospective, randomized, double-blind, placebo-controlled study are expected to be announced later this year (NCT04381377). However, to the best of our knowledge, there are no published studies specifically evaluating mentioned immune modulators in relation to immune parameters and lymphocytes exhaustion specifically in patients with COVID-19. Although we think, that used immune modulators could potentially have an impact on results of immune profile and lymphocytes exhaustion, none of our patients received these medications prior to the first sample collection. However, parameters examined longitudinally might be influenced. As the study has a retrospective character and it was not our aim to evaluate the effect of various immune modulators on examined immune parameters, different combinations of these medications, different dosing schedules and, importantly, different timing of their implementation to the treatment protocol during the course of the disease were used among our patients. Due to these reasons, it was not possible to evaluate, whether they have or have not influenced results of examined parameters or the outcome of our patients. More studies, designed to systematically assess effect of these medications on patients’ outcome and immune parameters (preferably randomized, double-blind, placebo-controlled studies) are needed to make conclusions.

Analysing the results of B-cell subsets phenotyping, we found only significantly higher proportion of switched-memory B-cells in asymptomatic and perished patients. Wang F. et al. (2020) did not find any significant differences in the percentage of memory B-cells (CD19^+^ CD27^+^) (Wang F. et al., 2020).

Neither our nor Song’s study (Song et al., 2020) found any significant difference in proportion of memory subsets of CD3^+^CD4^+^ cells between mild and severe cases. Unlike our results, Quin et al. (2020) detected significantly higher proportion of naïve CD3^+^CD4^+^ cells and significantly lower proportion of memory CD3^+^CD4^+^ cells in the group of severe cases (Quin et al., 2020). In contrast to these results, Wang F. et al. (2020) informed about increasing expression of CD45RO on CD3^+^CD4^+^ T-cells with increasing severity of COVID-19 disease (Wang F. et al., 2020). Discrepancies might be caused by different definition of severity, different stage of the disease captured by various authors, as well as unequal sample size.

Not many studies report the levels of immunoglobulins or complement in COVID-19 patients, probably because they did not significantly differ from reference values or among different severity groups (Jesenak et al., 2020). Our results suggest an evident, but non-significant decrease of IgA and IgG in the group of critically ill patients. Whether this was an accidental finding remains to be clarified. A Chinese study carried by Quin et al. (2020) did not show any significant differences in the serum levels of immunoglobulin isotypes nor complement proteins that were within normal range in COVID-19 patients. However, at the same time, they pointed to slight decrease of IgM in the group of patients with more severe COVID-19 disease (Quin et al., 2020). A research paper by Matricardi et al. (2020) suggested possible pathological role of high concentration of specific antibodies against SARS-CoV-2 in COVID-19 patients, promoting development of circulating immune complexes and subsequent enhancement of the macrophage activation syndrome (Matricardi et al., 2020). Soresina et al. (2020) and Quinti et al. (2020) presented few cases of patients with primary antibody deficiency, who, although developing pneumonia, did not require oxygen and had less severe course of COVID-19 disease compared to patients without antibody deficiency (Soresina et al., 2020; Quinti et al., 2020).

In our study, patients in group C and D did not significantly differ in basic parameters of specific cell immunity. Thus, (although specific antibodies were not examined and patients did not have primary antibody deficiencies), we speculate, that lower antibody level might correlate with lower ability of specific antibody production and this could be protective for this group of patients compared to those who died.

Even if we did not find any difference in the levels of C3 and C4, He et al. (2020) described possible regulatory effect of increased level of C3 in severe cases of COVID-19 disease (He et al., 2020).

The presence of circulating autoantibodies and development of autoimmune disorders in a subgroup of patients with severe COVID-19 disease has been confirmed. It was suggested, that SARS-CoV-2 virus can trigger autoimmunity in genetically predisposed individuals and produced autoantibodies might have a role in the pathophysiology of the disease (Halpert and Shoenfeld, 2020; Zhou et al., 2020). Autoantibodies against type I-IFN can be responsible for decreased ability to clear the virus (Bastard et al., 2020). A Kawasaki-like disease, known as MIS-C (in children) (Nakra et al., 2020) and MIS-A (in adults) (Sokolovsky et al., 2021), was described as a possible complication of COVID-19. Potential autoimmune complication might have had an impact on lymphocyte subsets as well as on markers of immune cells activation and exhaustion. Together 5 of our patients (across different severity groups) suffered from autoimmune disorders prior to SARS-CoV-2 infection (rheumatoid arthritis, myasthenia gravis, autoimmune thyroiditis) (**Table 1**). Unfortunately, we did not examine circulating autoantibodies, however none of our patients is known to develop obvious autoimmune complication neither MIS-A and there is no evidence of complement consumption in our patients.

Among limitations of this study, we should mention that it is a retrospective clinical observation of only a limited number of patients in different severity groups. The subgroups are unbalanced, there is no control group. Many findings have to be supported by further research but could be promising for targeted treatment of COVID-19.

Immune system and the changes in its reactivity play an evident role in various aspects of the COVID-19 pathology, from the increased susceptibility to infection in general, to the modulation of the clinical course and determining the clinical outcome of the disease. Besides the suppression of the absolute counts of various immune cells in the initial phase of COVID-19, their restoration could signal the clinical improvement. Another interesting issue is the (at least) temporary immune paralysis (exhaustion) assessed by the measurement of different surface markers on the immune cells. The disappearance of the expression of these markers could serve not only as a prognostic tool, but also as the potential therapeutic target especially in the early phases of COVID-19. Based on our results, we were able to observe an interesting potential of immune markers for monitoring the disease course and establishing a prognosis. We would like to analyse this observation in a larger number of well-defined COVID-19 patients.

## Data Availability Statement

The raw data supporting the conclusions of this article will be made available by the authors, without undue reservation.

## Ethics Statement

The studies involving human participants were reviewed and approved by Ethical Committee of University Hospital in Martin. The patients/participants provided their written informed consent to participate in this study.

## Author Contributions

AB was involved in literature search, collection, analysis and interpretation of the data, drafting and revising the manuscript. JP was involved in flow cytometric analysis, analysis of the data and in drafting the manuscript. RV was involved in drafting, revising and editing the manuscript. IK was involved in revising and editing the manuscript. LK was involved in literature search, drafting and revising the manuscript. MB was involved in flow cytometric analysis and analysis of the data and in drafting the manuscript. ZD was involved in literature search, drafting and revising the manuscript

MJ was involved in literature search, analysis and interpretation of the data, drafting and revising the manuscript. All authors contributed to the article and approved the submitted version.

## Funding

This study was co-funded by the project VEGA 1/0310/18 and has been produced with the support of the Integrated Infrastructure Operational Program for the project: Creation of a Digital Biobank to support the systemic public research infrastructure, ITMS: 313011AFG4, co-financed by the European Regional Development Fund.

## Conflict of Interest

The authors declare that the research was conducted in the absence of any commercial or financial relationships that could be construed as a potential conflict of interest.
